# AI and High-Grade Glioma for Diagnosis and Outcome Prediction: Do All Machine Learning Models Perform Equally Well?

**DOI:** 10.3389/fonc.2021.601425

**Published:** 2021-11-23

**Authors:** Luca Pasquini, Antonio Napolitano, Martina Lucignani, Emanuela Tagliente, Francesco Dellepiane, Maria Camilla Rossi-Espagnet, Matteo Ritrovato, Antonello Vidiri, Veronica Villani, Giulio Ranazzi, Antonella Stoppacciaro, Andrea Romano, Alberto Di Napoli, Alessandro Bozzao

**Affiliations:** ^1^ Neuroradiology Service, Department of Radiology, Memorial Sloan Kettering Cancer Center, New York, NY, United States; ^2^ Neuroradiology Unit, Neuroscience, Mental Health and Sensory Organs (NESMOS) Department, Sant’Andrea Hospital, La Sapienza University, Rome, Italy; ^3^ Medical Physics Department, Bambino Gesù Children’s Hospital, Scientific Institute for Research, Hospitalization and Healthcare (IRCCS), Rome, Italy; ^4^ Neuroradiology Unit, Imaging Department, Bambino Gesù Children’s Hospital, Scientific Institute for Research, Hospitalization and Healthcare (IRCCS), Rome, Italy; ^5^ Unit of Health Technology Assessment (HTA), Biomedical Technology Risk Manager, Bambino Gesù Children’s Hospital, Scientific Institute for Research, Hospitalization and Healthcare (IRCCS), Rome, Italy; ^6^ Radiology and Diagnostic Imaging Department, Regina Elena National Cancer Institute, Scientific Institute for Research, Hospitalization and Healthcare (IRCCS), Rome, Italy; ^7^ Neuro-Oncology Unit, Regina Elena National Cancer Institute, Scientific Institute for Research, Hospitalization and Healthcare (IRCCS), Rome, Italy; ^8^ Department of Clinical and Molecular Medicine, Surgical Pathology Units, Sant’Andrea Hospital, La Sapienza University, Rome, Italy; ^9^ Radiology Department, Castelli Romani Hospital, Rome, Italy

**Keywords:** glioblastoma, machine learning, radiomics, survival, high-grade glioma (HGG), genetics

## Abstract

Radiomic models outperform clinical data for outcome prediction in high-grade gliomas (HGG). However, lack of parameter standardization limits clinical applications. Many machine learning (ML) radiomic models employ single classifiers rather than ensemble learning, which is known to boost performance, and comparative analyses are lacking in the literature. We aimed to compare ML classifiers to predict clinically relevant tasks for HGG: overall survival (OS), isocitrate dehydrogenase (IDH) mutation, O-6-methylguanine-DNA-methyltransferase (MGMT) promoter methylation, epidermal growth factor receptor vIII (EGFR) amplification, and Ki-67 expression, based on radiomic features from conventional and advanced magnetic resonance imaging (MRI). Our objective was to identify the best algorithm for each task. One hundred fifty-six adult patients with pathologic diagnosis of HGG were included. Three tumoral regions were manually segmented: contrast-enhancing tumor, necrosis, and non-enhancing tumor. Radiomic features were extracted with a custom version of Pyradiomics and selected through Boruta algorithm. A Grid Search algorithm was applied when computing ten times K-fold cross-validation (K=10) to get the highest mean and lowest spread of accuracy. Model performance was assessed as AUC-ROC curve mean values with 95% confidence intervals (CI). Extreme Gradient Boosting (xGB) obtained highest accuracy for OS (74,5%), Adaboost (AB) for IDH mutation (87.5%), MGMT methylation (70,8%), Ki-67 expression (86%), and EGFR amplification (81%). Ensemble classifiers showed the best performance across tasks. High-scoring radiomic features shed light on possible correlations between MRI and tumor histology.

## Introduction

High-grade gliomas (HGG) are considered the most frequent and lethal primary malignant brain tumors of the adult (1). Glioblastoma multiforme is a type of HGG with an estimated incidence rate of 3.19 per 100,000 persons in the United States, a median age of 64 years, and a dismally poor overall survival (OS) despite combined radio-chemotherapy, ranging approximately between 15 and 17 months (1, 2). Although less frequent, the outcome of HGG is similarly poor in the pediatric population (3). Genetic alterations may influence patient outcome, with effects on survival, disease progression, and treatment response (2, 4). These considerations inspired the cIMPACT recommendations for classification of diffused gliomas and the last revision of the World Health Organization (WHO) classification for central nervous system (CNS) tumors, which suggested considering isocitrate dehydrogenase (IDH)-mutant and IDH-wild-type cancers as two separate entities due to the importance of IDH mutation for patient survival (5, 6).

Artificial intelligence (AI) is the term used to describe the use of computers and technology to simulate intelligent behavior and critical thinking comparable to a human being. Specifically, machine learning (ML) is a subfield of AI, defined as a set of methods that can automatically detect a pattern of data, with the ability of using uncovered patterns to predict future data or perform other kinds of decision-making under uncertainty (7). The learning process can be classified as supervised and unsupervised. Unsupervised learning models identify the pattern class information heuristically, providing clusters without a ground-truth knowledge. On the contrary, the supervised learning approach (explored in this article) identifies a pattern that connects the inputs X to the outputs Y, given a labeled set of input-output pairs. In recent years, AI applications in medicine have grown exponentially, involving almost every medical specialty (8). In the field of radiology, the conversion of biomedical images [such as magnetic resonance imaging (MRI), Computerized Tomography (CT), X-Ray, etc.] to mineable data, and their analysis with AI techniques is defined as “radiomics” (9). Thanks to these new developments, it is possible to extract multiple features from radiological images reflecting tissue characteristics, and use them as input for ML models. For example, graytone distribution and mutual dependencies reflect tissue heterogeneity (10). One of the most interesting applications of ML to radiology is the creation of predictive models to estimate clinically relevant variables. Biomedical images intrinsic parameters (represented by radiomic features) contain information about tissue structure, molecular data, and patient outcome, providing important information for patient care through quantitative image analyses (9, 11). AI-powered analyses may aid diagnosis and prognostication, with practical applications in multiple clinical settings, including emergency care (12).

In brain tumors, radiomic research can identify features that describe the tumor microenvironment (13) and build predictive models for tumor variables and patient outcome. Radiomic models have been shown to outperform clinical models based on patient age, Karnofsky performance scale, surgical resection, genetic alterations, in glioblastoma (GBM) outcome prediction (14, 15). Recent studies proposed several high-performance radiomic models for predicting OS, progression-free survival, molecular subtypes of HGG, as well as genetic alterations critical for clinical practice (16–20). Despite these promising results, clinical implementation is extremely limited due to wide variations of model performances (21–23) and controversial findings. For example, a recent study on 152 patients with GBM concluded that MRI features were not adequate for providing reliable and clinically meaningful predictions through ML classification models (24). A recent review calls for improved standardization and clinical application feasibility (25).

Variability in model performance may depend on parameters optimization. Radiomic workflows comprehend multiple steps requiring parameter choice: tumor segmentation on radiologic images to identify regions of interest (ROIs), feature extraction and selection, training, testing and validation of the AI model, performance evaluation (26, 27). The lack of radiomic parameters standardization might limit results generalizability across studies. A possible solution for this limitation is to compare multiple ML algorithms in the same population for different tasks. In fact, the classification method was shown to be the dominant source of performance variation in radiomic analyses (28). Furthermore, most of radiomic models presented for outcome prediction in HGG employ classic ML algorithms, such as logistic regression, support vector machine, and decisional trees (21, 22). Non-ensemble learners showed inferior performance for small or imbalanced datasets when compared to the ensemble counterpart. Few studies have indeed shown comparative results of single learners *vs* ensemble models (29–31). This is not unexpected considering that single classifier approaches try to learn a single hypothesis from the training set, whereas ensemble learning tries to construct a set of hypotheses and combine them in the best way possible (32). In fact, ensemble methods are used to obtain better predictive performance by reducing both the bias (representational problem) and the variance (computational problem) of learning algorithms (33).

In this study, we chose well-established ML classifiers from previous literature in the field and compared their performance to predict outcome variables of HGG: OS, IDH mutation, O-6-methylguanine-DNA-methyltransferase (MGMT) promoter methylation, epidermal growth factor receptor vIII (EGFR) amplification, and Ki-67 expression, based on features extracted from conventional and advanced MRI. Our objectives were (1) to assess the best algorithm for each prediction task, providing a benchmark for future clinical applications. Particularly, we wanted to compare classic and ensemble learners among ML classifiers to provide a comprehensive view on model performance; (2) to evaluate highly predictive radiomic features extracted from different tumor regions, highlighting possible correlations between MR parameters and tumor molecular/genetic characteristics.

## Materials and Methods

### Subjects

This retrospective observational study was conducted in accordance to the Helsinki declaration. Approval from the institutional review board (IRB) was obtained with protocol number: 19 SA_2020. Consecutive patients with pathologically proven diagnosis of HGG were recruited from March 2005 to May 2019. Data were collected from two institutions: Sant’Andrea Hospital La Sapienza University of Rome (Institution 1) on a 1.5T scanner (Magnetom Sonata, Siemens, Erlangen, Germany), and Regina Elena Institute of Rome (Institution 2) on a 3T system (Discovery MR 750w, GE Healthcare, Milwaukee, WI, USA). We enrolled patients fulfilling the following inclusion criteria: histopathological diagnosis of HGG, presurgical MRI with at least one sequence among structural T1 or T2-weighted images, diffusion or perfusion-weighted images. Exclusion criteria were causes of suboptimal images (for example motion artifacts) and loss of patients’ information during follow-up.

All patients received standard treatment after surgery with the same protocol, including focal radiotherapy (RT) and concomitant temozolomide (TMZ), followed by adjuvant TMZ therapy. RT consisted of fractionated focal irradiation (60 Gy) started within 4 weeks after surgery. The radiation dose was delivered in 30 fractions of 2 Gy over 6 weeks. Chemotherapy with TMZ was administered in a dose of 75 mg/m2, 7 days/week. Adjuvant TMZ started 4 weeks after radiation with the following protocol: 150 mg/m2 for the first cycle, increased to 200 mg/m2 for the second cycle; administered 5 days every 28 days up to 12 cycles.

Prediction labels were associated with survival at 12 months after diagnosis (SURV12), MGMT promoter methylation, IDH mutation, Ki-67 expression, and EGFR amplification. These labels were chosen as they usually provide important prognostic information in HGG. Survival cutoff at 12 months was set based on previous studies (34–36).

### Histopathological Analysis

Each tumor specimen was fixed in formaldehyde (10%) and embedded in paraffin. Thin sections (2 μm) were mounted and stained with hematoxylin and eosin. The histopathological examination, including tumor grading, was performed taking into account at least three of the following: cellular atypias, number of mitotes, microvascular proliferation, and/or presence of necrosis. The histopathological examination was performed according to the 2016 edition of the WHO classification of CNS tumors.

### Immunohistochemistry

A Dako Envision Flex system was employed for the immunohistochemical analysis. The immunostaining patterns of EGFR were evaluated considering both cellular and tissue distribution. The number of immunopositive cells in 10 high-power (40×) areas were counted, and the percentage of immunopositive cells were estimated. The ratio of positive cells/total number of cells was calculated for each field. The mean value of the 10 fields obtained from a section was considered as the estimated percentage of immunoreactivity assigned to the tumor sample. For IDH-1 mutation analysis, we performed a test with IDH-1 R132H antibody. A positive result was defined when a focal or diffuse immunopositivity was detected, while a negative result was when no immunopositive tumor cells were found. Negative cases were further analyzed for IDH-1/2 mutations as previously shown (37). All sequence reactions were carried out using the GenomeLab DTCS quick-start kit (Beckman Coulter, Fullerton, CA, USA). The reactions were carried out in an automated DNA analyzer (CEQ 8000; Beckman Coulter). All sections were immunostained with Ki-67 antibody. The positivity for Ki67 was determined by counting at least 1,000 tumor cells in a homogeneously stained area and then expressed in percentage.

### MGMT Methylation Testing

We used EntroGen’s MGMT Methylation Detection Kit (MSPCR, Cat. No. MGMT-RT44), a semiquantitative real-time PCR-based essay for detection of MGMT promoter methylation within the DMR2 locus, distinguishing between methylated and non-methylated cytosines. Its target region starts at chr10:131265513 (hg19 genome build) in the MGMT promoter region and covers CpG sites 75–86. The detection of the amplification product was done by using fluorescent hydrolysis fraction. The procedure involves the following steps: (1) isolation of DNA from tumor biopsies, paraffin-embedded sections; (2) bisulfite treatment of the isolated DNA using the EZ DNA methylation-Lightning Kit (Zymo Research, CATD5030); (3) amplification of treated DNA using the provided reagents in the MGMT Promoter methylation Detection kit; (4) data analysis and interpretation using the real-time PCR software.

### MRI Acquisition

MRI sequences were acquired with the same protocol including magnetization-prepared rapid acquisition with gradient echo (MPRAGE), fluid-attenuated inversion recovery (FLAIR), T1-weighted, T2-weigthed, diffusion weighted images (DWI), with apparent diffusion coefficient (ADC) map reconstruction, and perfusion weighted images (PWI) with dynamic susceptibility contrast (DSC) technique. Perfusion parametric maps were obtained through a dedicated software package OleaSphere software version 3.0 (Olea Medical, La Ciotat, France). A relative cerebral blood volume (rCBV) map was generated by using an established tracer kinetic model applied to the first-pass data (38). As previously shown (39), we applied a mathematical correction to the dynamic curves to reduce contrast agent leakage effects. Detailed acquisition parameters can be found in the **Supplementary Material**.

### Image Processing and Radiomic Feature Extraction

**Figure 1:**
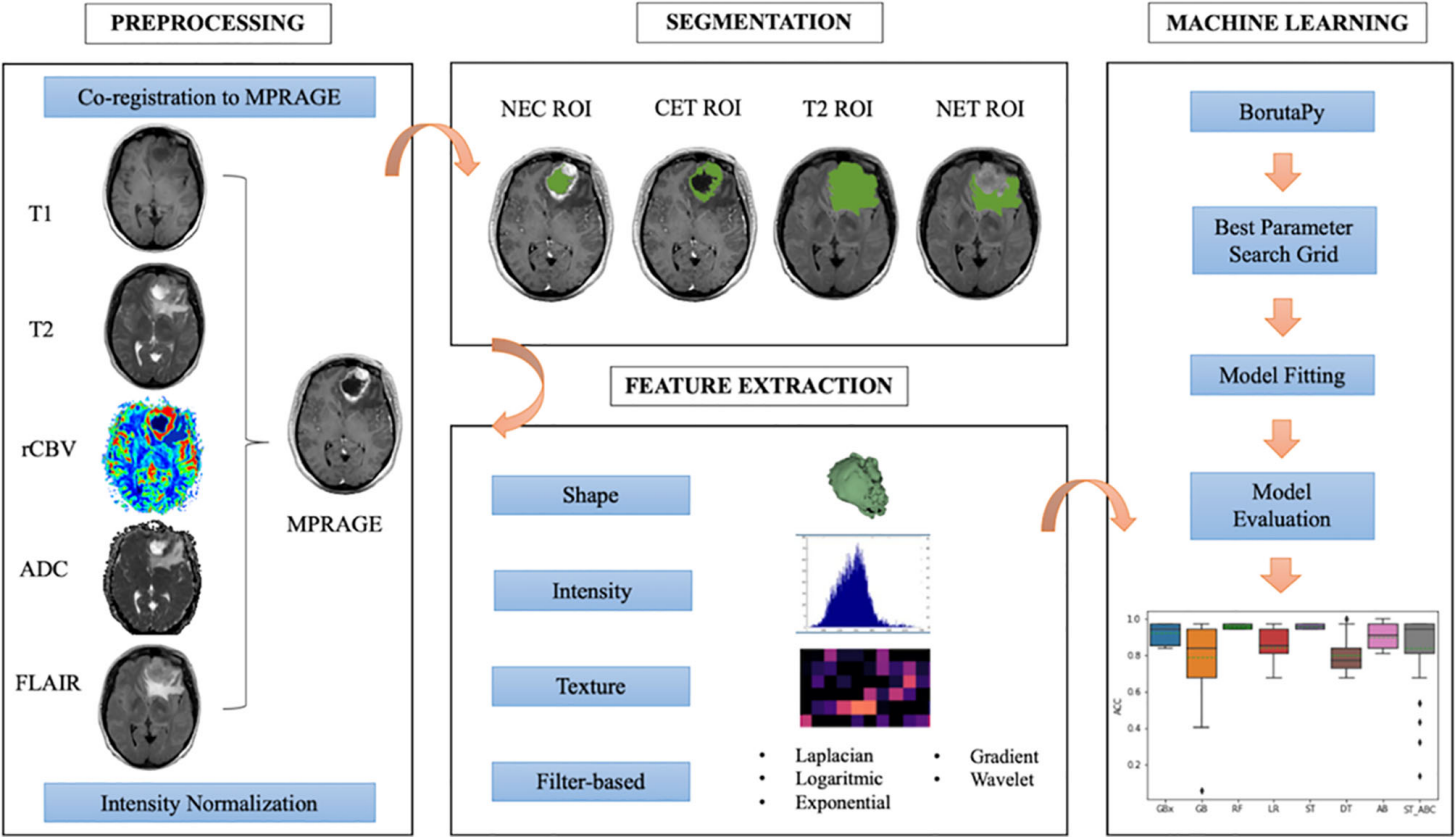
Radiomic workflow followed in the present study.

The radiomic workflow of our analysis was developed following the white paper of the Image Biomarker Standardization Initiative (IBSI) (40) and is summarized in **Figure 1**. For every patient, we automatically co-registered MR data to the MPRAGE sequence using FMRIB Linear Image Registration Tool of FSL (https://fsl.fmrib.ox.ac.uk) (41, 42). Tumors were manually segmented by a neuroradiologist, with three ROIs drawn on MPRAGE and FLAIR images using 3D-Slicer (LP, with 7 years of experience in radiology) (https://www.slicer.org/) (43). Doubtful cases were solved as for consensus with a senior neuroradiologist (AB, with 25 years of experience in radiology). The ROIs were whole tumor (T2), contrast-enhancing tumor (CET), necrosis (NEC). A further non-enhancing tumor (NET) ROI was obtained from the other ROIs as it follows: T2 – (CET+NEC). Based on recent findings (44), we performed intensity non-standardness correction on our multi-institutional data by scaling each image with respect to its mean value within specific brain structure (i.e., NET ROI) using MATLAB R2017a environment (MATLAB 2017, Natick, MA, USA: The MathWorks Inc). The intensity range between 0 and 255 was not rescaled to prevent information loss due to image down-sampling.

We extracted a set of 1,871 radiomic features for each patient from the combination of tumor ROIs (NET, CET, and NEC) and multiparametric MR data (ADC, FLAIR, MPRAGE, rCBV, T1-weigthed, and T2-weighted images). The process was carried out through Pyradiomics package on Python 2.7 (45). Each radiomic set included 14 shape features, 18 intensity features, and 75 texture features [gray-level co-occurrence matrix (GLCM), gray-level difference matrix (GLDM), gray-level size zone matrix (GLSZM), gray-level run length matrix (GLRLM), neighborhood gray tone difference matrix (NGTDM)] from original and filtered images (wavelet decomposition, Laplacian of Gaussian, exponential, logarithmic, and gradient). Additionally, three *ad-hoc* fractal features were computed: box counting two dimensions (2D), box counting three dimensions (3D), and differential box counting, which were integrated in the code of the Pyradiomics pipeline (46). Patients’ age at the time of diagnosis was considered a feature in our model for survival prediction only.

### Feature Selection and Classification

The pipeline was written in Python and was implemented on Google Colab (47). Prior to any further analysis, each extracted feature distribution was standardized by taking out outliers, removing the mean and scaling it to unit variance with Python Standard Scaler package. Feature selection was then performed in order to identify an ensemble of the most predictive features for each ROI-sequence combination. To this purpose, we used the Boruta algorithm, a powerful and recently introduced feature selector method, that trained a Random Forest Classifier on a duplicated dataset (composed by original and shadow features) and marked a feature as important comparing its Z-scores with that of the duplicate (48). The implementation we used in this work was boruta_py module, freely accessible from github repository (49). Due to the retrospective nature of this study, some MRI sequences were not acquired for all the patients, and some patients lacked full genetic testing, leading to class imbalance issues. In order to overcome this limitation in binary classification, we used Synthetic Minority Over-sampling Technique (SMOTE) approach, which oversamples data of the minority class, creating new synthesized samples from the existing ones (24, 50).

**Figure 2:**
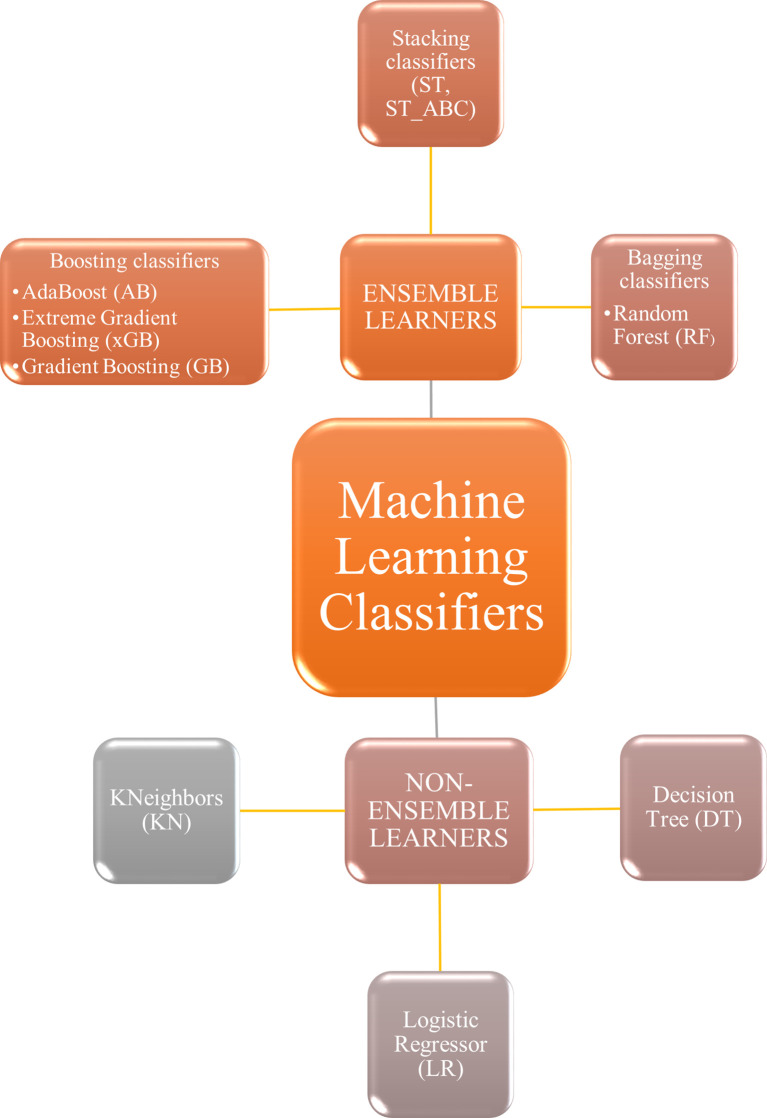
Machine learning classifiers tested in the present study. Non-ensemble learners included KNeighbors, logistic regressor, and decision tree. Ensemble learners included boosting, stacking, and bagging classifiers.

To find the best parameter setting, an optimization search grid algorithm was applied on nine ML classifiers including ensemble and non-ensemble learners (**Figure 2**): AdaBoost (AB), Extreme Gradient Boosting (xGB), Gradient Boosting (GB), Decision Tree (DT) and Random Forest (RF), Logistic Regressor (LR), two types of Stacking classifiers: stacking (ST) and stacking with AdaBoost (ST_ABC), and KNeighbors (KN). AB, xGB, and GB use a set of weak learners and try to boost them into strong learners. The GB classifier appears in classification studies (24), as it works well with categorical and numerical data; we decided to compare GB performance with xGB, that is the fastest implementation of gradient boosted trees (24, 51). The AB was also often used for brain tumor classification (52, 53), as it works to create a powerful algorithm where instances are reweighted rather than resampled. A Decision Tree algorithm was used in AB as a weak learner. Decision Tree (DT) and Random Forest (RF) are both based upon decision tree algorithms. RF is actually a collection of DTs attempting to classify a new object based on its attributes (54). The RF classifier was already used in brain tumor segmentation problems (55), for the MGMT promoter prediction model (56), for the IDH status prediction (57), and for the survival prediction (58). Logistic Regressor (LR) is one of the most used linear classifiers to disentangle linear relationship between the data (24). The stacked generalization is an ensemble ML algorithm that learns how to best combine the predictions from multiple well-performing ML models. In our case, one classifier was set on the best parameters from GB, RF, and LR (ST), whereas the second was set on best parameters from GB, RF, and AB (ST_ABC) (59). KN relies on distance in data space and is one of the simplest of all the supervised ML algorithms (31). Apart from the extreme gradient boosting classifier which was implemented in xgboost package (60), all classifiers were part of Scikit-learn package (61). Algorithms were chosen based on their known performance and extensive use in the literature.

In order to achieve the most performant and robust model, the Grid Search algorithm, as implemented in Scikit-learn package, was applied when computing 10 times K-fold cross-validation (K=10) and setting the same test split. Given the unbalanced condition for all molecular predictors and in order to reach the same number of trials as for SURV12, an iterative way of K-fold cross-validation was applied. This method made sure that among the possible combinations of data splitting, only those one having the number of minority class subjects at least equal to half of the number of majority class were included among the eligible reshuffles. The Grid Search algorithm was set to look for the highest mean along with the lowest spread of accuracy. The accuracy mean and standard deviation were evaluated on 100 different splitting of training and test data. Once optimal parameters were identified, model performances were also assessed in terms of AUC-ROC curve with 95% CI (28, 62). AUC-ROC curves were also useful when comparing classifiers as they show the trade-off between false positive and true positive rates in the classification (63).

## Results

### Subjects

The study included 156 adult patients (mean age = 62 y, range = 35–83 y) with confirmed diagnosis of HGG: 121 patients were acquired at Institution 1 and 35 patients at Institution 2. Descriptive statistics performed on genetic variables revealed an odds ratio of 0.607, 1.186, 0.911, and 5.6 for Ki-67, MGMT, IDH, and EGFR respectively, evaluated with reference to SURV12.

### Machine Learning Analysis

The distribution of our data is summarized in **Table 1**. For those labels suffering from class imbalance issues, SMOTE was always used. Feature selection produced multiple radiomic signatures composed by 20 features, ordered by importance for the predicted label. The best 15 features for every signature are displayed in the **Supplementary Material**. Nine ML classifiers were compared in the present study. We identified the best classifier and the best ROI-sequence combination in terms of prediction accuracy for each task (SURV12, MGMT, IDH, KI67, and EGFR).

**Table 1:** Number of patients and label distributions for label-sequence combination.

	ADC	FLAIR	MPRAGE	rCBV	T1	T2
SURV12 (0/1)	134 (65/69)	140 (68/72)	138 (66/72)	93 (45/48)	122 (61/61)	122 (60/62)
MGMT (0/1)	110 (41/69)	115 (43/72)	114 (42/72)	80 (33/47)	100 (39/61)	102 (39/63)
IDH (0/1)	86 (71/15)	89 (74/15)	89 (74/15)	60 (51/9)	77 (63/14)	78 (65/13)
KI67 (0/1)	100 (18/82)	106 (21/85)	103 (22/81)	77 (16/61)	97 (17/80)	94 (16/78)
EGFR (0/1)	65 (21/44)	69 (23/46)	66 (23/43)	49 (16/33)	65 (22/43)	62 (20/42)

### Prediction Performance

**Figure 3:**
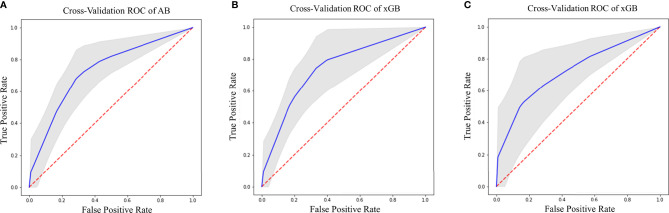
Best ROC curves for Surv12 prediction: **(A)** AB classifier with ADC sequence on NET ROI; **(B)** xGB classifier with T2 sequence on NEC ROI; **(C)** xGB classifier with FLAIR sequence on NET ROI.

Regarding SURV12 prediction, the best performance was achieved by AB and xGB classifiers on ADC radiomic features from NET ROI and T2 radiomic features from NEC ROI (**Table 2**). AB classifier demonstrated accuracy of 73.6% and AUC-ROC mean value of 73.6% (95% CI 71.6–75.3) based on ADC features from NET ROI (**Figure 3A**). xGB classifier achieved accuracy of 74.5% and AUC-ROC mean value of 74.2% (95% CI 71.9–76.3) with T2 radiomic features from NEC ROI (**Figure 3B**). Similarly, xGB classifier provided good accuracy based on FLAIR features from NET ROI (Acc=72.1%; AUC-ROC=72.4%; 95% CI 69.6–75) (**Figure 3C**).

**Table 2:** Surv12 best results (reported as mean ± standard deviation).

ROI	SEQ		xGB	GB	RF	LR	ST	KN	DT	AB	ST_ABC
NET	ADC	Acc%	71,8 ± 10	68,8 ± 11,4	67,9 ± 6,5	46,3 ± 5,4	71 ± 9	61,2 ± 12,3	59,2 ± 11,7	73,6 ± 9,3	64,2 ± 12,6
NET	ADC	Roc %	71,8 ± 9,7	69,1 ± 11,1	67,9 ± 6,5	46,3 ± 5,4	71 ± 9	61,2 ± 12,3	59,2 ± 11,7	73,6 ± 9,3	64,2 ± 12,6
NET	FLAIR	Acc %	72,1 ± 13,7	67,4 ± 9,9	71,6 ± 8,4	62 ± 13,6	69 ± 12	54,3 ± 15	59 ± 13,7	68,9 ± 7	62,3 ± 14
NET	FLAIR	Roc %	72,4 ± 14	67 ± 11	72,1 ± 7,6	62,3 ± 13,7	69 ± 12,2	53,9 ± 14,8	58,8 ± 13	69,5 ± 7,7	59 ± 13
NEC	T2	Acc %	74,5 ± 11	65,8 ± 12,6	67 ± 16,7	58,7 ± 14,3	73,6 ± 9	52,3 ± 15,2	60,7 ± 11,4	72,7 ± 9,5	58,1 ± 13,9
NEC	T2	Roc %	74,2 ± 10,9	65 ± 11,2	66,4 ± 17	58,8 ± 14,4	73 ± 9,4	52 ± 14,9	59 ± 11	72,5 ± 9,6	56,3 ± 14,3

**Figure 4:**
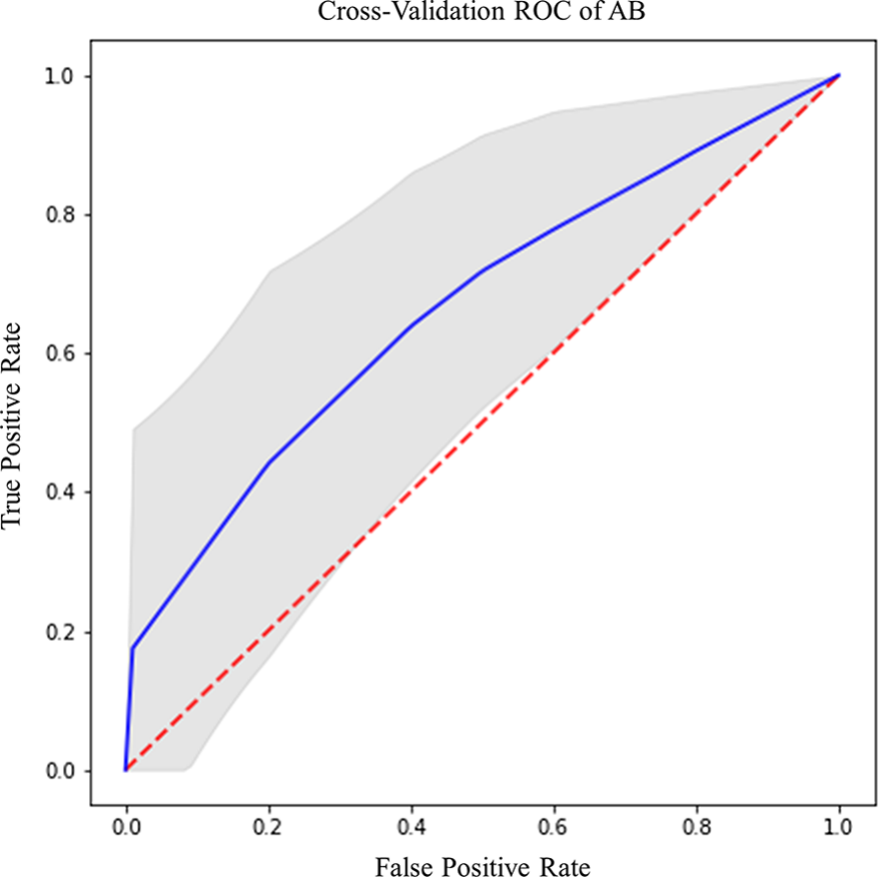
Best ROC curve for MGMT prediction: AB classifier with FLAIR sequence on CET ROI.

Best results for MGMT prediction (**Table 3**) were obtained from CET ROI on FLAIR images by using AB classifier (Acc=70.8%; AUC-ROC=68.8%; 95% CI 65.9–71.7) (**Figure 4**). High-scoring features mainly included texture parameters (**Figure S4**).

**Table 3:** MGMT best results (reported as mean ± standard deviation).

ROI	SEQ		xGB	GB	RF	LR	ST	KN	DT	AB	ST_ABC
CET	FLAIR	Acc %	63,3 ± 11,3	68,1 ± 13,4	70.7 ± 9,3	65,5 ± 11,4,4	67,9 ± 15,7	52,2 ± 12,7	59,4 ± 14,4	70,8 ± 14,1	64,5 ± 15,7
CET	FLAIR	Roc %	62,8 ± 11,7	66,8 ± 13,4	63,4 ± 12,2	59 ± 10,6	67 ± 16,8	51,4 ± 13,3	55,5 ± 12,1	68,8 ± 14,6	62 ± 14,2

**Figure 5:**
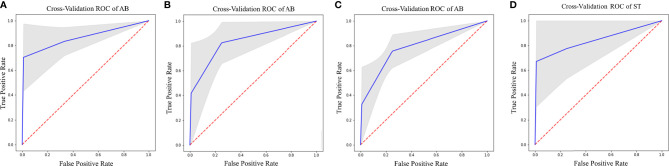
Best ROC curves for IDH prediction: **(A)** AB classifier with rCBV sequence on NET ROI; **(B)** AB classifier with T2 sequence on CET ROI; **(C)** AB classifier with T2 sequence on NEC ROI; **(D)** ST classifier with T1 sequence on NET ROI.

IDH prediction task showed the best performance in our dataset (**Table 4**). Highest accuracy was achieved by AB classifier with rCBV features from NET ROI (Acc= 87.5%; AUC-ROC=86.7%; 95% CI 84.3–89) (**Figure 5A**). Similarly, AB classifier provided good results with T2-based features from CET ROI (Acc=85.9%; AUC-ROC=85.8%; 95% CI 80–84.6) (**Figure 5B**) and NEC ROI (Acc=80.8%; AUC-ROC=80.5%; 95% CI 78.4–82.6) (**Figure 5C**). Good results were also achieved by ST classifier based on T1 features from NET ROI (Acc=84.2%; AUC-ROC=83%; 95% CI 80–85.9) (**Figure 5D**).

**Table 4:** IDH best results (reported as mean ± standard deviation).

ROI	SEQ		xGB	GB	RF	LR	ST	KN	DT	AB	ST_ABC
NET	rCBV	Acc %	83,5 ± 12,8	82,8 ± 12	76,2 ± 16,2	77,3 ± 14,4	86,7 ± 11,8	69,2 ± 17,5	78,7 ± 14,5	87,5 ± 11,9	82,8 ± 12,4
NET	rCBV	Roc %	83,2 ± 12,8	82 ± 13,5	78,3 ± 15,5	78 ± 14,7	85,8 ± 12,3	69 ± 18,3	78,3 ± 15	86,7 ± 12	82 ± 12,4
NET	T1	Acc %	80,2 ± 14	81 ± 13,8	80 ± 12,5	68,7 ± 12	84,2 ± 15	66 ± 21	75,2 ± 13,7	85,9 ± 14	80,9 ± 12
NET	T1	Roc %	79,4 ± 15	80,7 ± 15	78,2 ± 12,3	67,9 ± 11,4	83 ± 14,7	66,7 ± 21,2	76,3 ± 14,5	85,8 ± 14,9	80 ± 13
CET	T2	Acc %	80,2 ± 14	81 ± 13,8	80 ± 12,5	68,7 ± 12	84,2 ± 15	66 ± 21	75,2 ± 13,7	85,9 ± 14	80,9 ± 12
CET	T2	Roc %	79,4 ± 15	80,7 ± 15	78,2 ± 12,3	67,9 ± 11,4	83 ± 14,7	66,7 ± 21,2	76,3 ± 14,5	85,8 ± 14,9	80 ± 13
NEC	T2	Acc %	77,4 ± 9,8	77,9 ± 11	79 ± 11	70,3 ± 12,5	79,2 ± 10,7	69,3 ± 14,3	75,8 ± 12,6	80,8 ± 10,2	79,5 ± 9,5
NEC	T2	Roc %	76,6 ± 10	77 ± 10	78 ± 11,2	70,7 ± 12,6	78,9 ± 9,7	70 ± 14,9	77,5 ± 12,9	80,5 ± 10,6	78,4 ± 9

**Figure 6:**
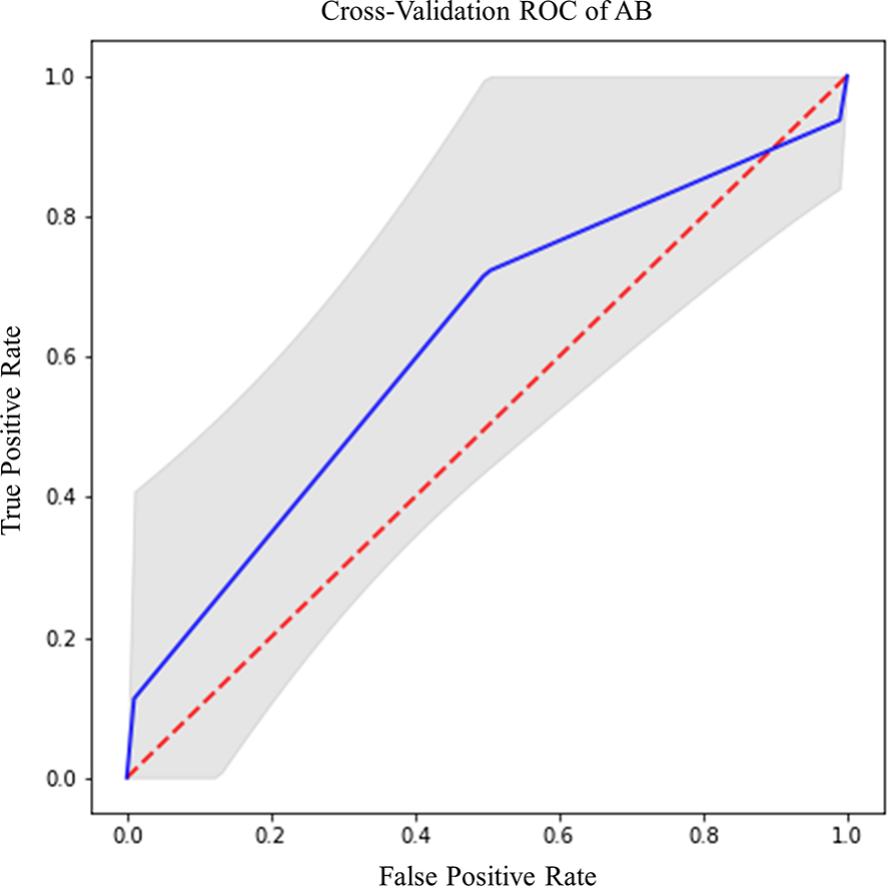
Best ROC curve for KI67 prediction: AB classifier with ADC sequence on CET ROI.

The prediction of Ki-67 expression provided excellent results from ADC sequence and CET ROI (**Table 5**). AB classifier provided the highest accuracy (86%) and AUC-ROC value (70%; 95% CI 65.3–72.9) (**Figure 6**).

**Table 5:** KI67 best results (reported as mean ± standard deviation).

ROI	SEQ		xGB	GB	RF	LR	ST	KN	DT	AB	ST_ABC
CET	ADC	Acc %	82,3 ± 8,4	81,6 ± 9,7	83,9 ± 9,8	63,7 ± 13,6	82,6 ± 10,5	67,5 ± 10	76,5 ± 12	86 ± 10,6	83 ± 8,2
CET	ADC	Roc %	64,6 ± 15	64,5 ± 17,3	67,5 ± 18,9	50,8 ± 17,5	63,2 ± 17,8	60 ± 15,7	60 ± 19	70 ± 20	64,4 ± 17

**Figure 7:**
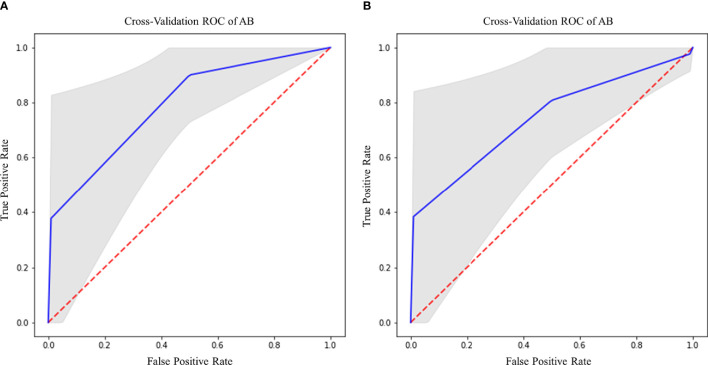
Best ROC curves for EGFR prediction: **(A)** AB classifier with rCBV sequence on CET ROI; **(B)** AB classifier with T2 sequence on CET ROI.

EGFR amplification was correctly predicted by radiomic features extracted from rCBV and T2 images within CET ROI, in both cases with AB classifier (**Table 6**). Particularly, rCBV demonstrated the highest performance (Acc=81%; AUC-ROC=74.3%; 95% CI 70.8–77.8) (**Figure 7A**), while T2 sequence achieved accuracy of 77.8% and AUC-ROC equal to 74.1% (95% CI 70.6–77.6) (**Figure 7B**).

**Table 6:** EGFR best results (reported as mean ± standard deviation).

ROI	SEQ		xGB	GB	RF	LR	ST	KN	DT	AB	ST_ABC
CET	rCBV	Acc %	69,8 ± 15,1	75,4 ± 15	73,1 ± 16	64,3 ± 16,3	72,9 ± 14,3	61,3 ± 21,4	66,7 ± 19,4	81 ± 13,8	66,5 ± 18,7
CET	rCBV	Roc %	63,9 ± 19,5	64,6 ± 18,5	64,7 ± 20	62,2 ± 21,8	65,7 ± 18,9	63,4 ± 23,3	59,4 ± 23,2	74,3 ± 17,3	62,6 ± 20
CET	T2	Acc %	76,4 ± 15,2	74,7 ± 15	76,4 ± 16	60,8 ± 18,8	76 ± 17,8	59,7 ± 20,4	61,3 ± 18,7	77,8 ± 13,8	71,5 ± 16
CET	T2	Roc %	70,4 ± 22,7	69,7 ± 19,8	76,3 ± 17	65,4 ± 15,7	69,8 ± 22,8	60,2 ± 19,5	55,7 ± 20,4	74,1 ± 17,6	65,6 ± 20,6

Box-plots figures comparing the best results for each classifier and tables with high-scoring radiomic features are provided in the **Supplementary Material** (**Figures S1**–**S10**).

## Discussion

AI has proven to be an accurate tool in predicting survival and molecular profile of gliomas. However, high variability in results across studies and lack of standardization are limiting its use in clinical practice. We studied the best ROI-sequence combination for prediction of clinically relevant variables in HGG, by comparing multiple ML classifiers including classic and ensemble learners. Ensemble classifiers achieved the best performance in every task. The AB was the best classifier overall, with accuracy of 73.6, 70.8, 87.5, 86, and 81% for SURV12, MGMT, IDH, Ki-67, and EGFR respectively, while the LR and KN classifiers always produced suboptimal prediction performances.

These results are in line with previous literature comparing boosting and logistic regression-based classifiers (64). Ensemble models showed high classification performance in different fields. Similar results were observed by Wang et al. using four single classifiers combined with three different algorithms (bagging boosting and stacking) to create ensemble learners for credit scoring (59). All ensemble types yielded a significant improvement compared to base learners (59). In line with our findings, Lu et al. reported higher performances for AdaBoost compared to bagging ensemble algorithms for cancer classification with gene expression data. The idea behind this better performance is that AdaBoost is based on a linear combination of single learners weighted by their own performance, being able to filter out redundant training data attributes and focusing on the important features (65).

Other studies compared ML classifiers in HGG, although with different methodologies and results. Samara et al. conducted a study comparing base models (LR, KN, DT, linear support vector machine) and ensemble algorithms (Bootstrap aggregating, AB, RF, and Voting classifier) in a GBM prognostication model based on clinical data (30). In the study, ensemble classifiers attained the highest AUC for every dataset, especially when trained on statistically determined sets or union sets. Osman attempted GBM patients’ survival stratification based on conventional MRI sequences with several classifiers. Combining nine selected radiomic features with clinical factors (e.g., age and resection status), even the best prediction accuracy of the ensemble learning classifier appeared low (less than 60%), possibly due to the multi-institutional nature of the study (31). In our approach, we made use of advanced sequences and a larger number of features. Among them we also included fractal dimension-based features which have rarely been implemented in previous studies and may help boosting up the accuracy of our results. Further and important difference regards the use of Boruta algorithm to reduce the features and select only those having higher importance for the model. Also, Kickingereder et al. proposed to evaluate the association of multiparametric MRI features with molecular characteristics (e.g., global DNA methylation subgroup, MGMT, EGFR) in GBM patients, training different models (e.g., stochastic GB, RF, and penalized LR). The authors found associations between established MRI features and molecular characteristics (prediction accuracy of 63% for EGFR with penalized LR). However, the link between them was not strong enough to enable generation of ML classification models for reliable and clinically meaningful predictions (24). In addition to a different set of predicted outcomes, this result might be due to the type and amount of imaging features used for prediction: Kickingereder et al. used 31 imaging parameters for molecular characteristic prediction, while this study extracted 1,871 radiomic features from each image.

A closer look on best performing features and ROI-sequence combinations from our results may unravel interesting associations between MRI parameters and pathologic features of HGG. The best survival prediction was achieved by AB using ADC maps from NET ROI. Also, xGB classifiers showed high performance using T2 images from NEC ROI or FLAIR images from NET ROI, but with higher spread of accuracy (**Table 2**). Previous studies showed heterogeneous results on the same matter (17, 31, 66), depending on size and source of datasets, type and number of extracted features, and model parameters. NET is a common finding in HGG and is considered a combination of infiltrating tumor cells and vasogenic edema (67), whose extension correlates with poor prognosis (68). After surgical resection, recurrence occurs more frequently along the resection margins, due to populations of malignant cells interspersed in the NET (69). Recent research demonstrated that peritumoral MRI textural features from FLAIR and T2 images were predictive of survival as compared to features from enhancing tumor, necrotic regions, and known clinical factors (70, 71). Higher performance of ADC features from NET is coherent with studies demonstrating the inverse correlation between ADC values and tissue cellularity (72–75). In fact, tissue cellularity as measured by ADC can differentiate between vasogenic edema and malignant tumoral tissue within the NET, possibly recognizing patients at higher risk for recurrence (76). Good survival predictivity on NEC ROI is also supported by previous literature. Chaddad et al. reported that shape features, particularly those extracted from necrotic regions, can be used to effectively predict OS of GBM patients (77). Furthermore, our best performing feature for survival prediction on NEC was related to fractal dimension (**Figure S2C**), a measure of shape complexity that has rarely been employed in radiomic studies but demonstrated interesting correlations with patient survival (35).

Preoperative prediction of MGMT promoter methylation and IDH mutation represents a crucial objective for radiomic studies due to their pivotal role in patient outcome (2, 4). On conventional and advanced MRI, MGMT methylated HGG may show mixed nodular enhancement, limited edema, lower rCBV, increased Ktrans, and higher ADC minimum values (78, 79). IDH mutant tumors usually show less enhancement, less blood flow on perfusion weighted images, higher mean diffusion values, smaller size, and frontal lobe location (21). Many studies tried to correlate these characteristics with MGMT and IDH status, reporting conflicting results (78). Textural features demonstrated higher accuracy for MGMT promoter methylation prediction, achieving best performance with FLAIR features from CET (70.8%, AB classifier) (**Figures S3** and **S4**). These results are coherent with other reports (80) and confirm that textural features outperform morphological and intensity features in MGMT status prediction (16). Another recent study from Sasaki et al. reported accuracy of 67% for MGMT prediction with textural features (81). A possible explanation for the performance discrepancy is the choice of the classification algorithm: prediction accuracy has great variability depending on the selected model (**Table 3**), with higher performance for ensemble learners. Regarding IDH mutation, our AB classifier achieved an accuracy of 87.5% with rCBV-derived first-order features (median, skewness) from NET (**Figure S6A**), outperforming most of previous models (21, 22). Besides correlating with patient survival (82), perfusion-based features were highly predictive of IDH status in another recent study from our group based on deep-learning (37). Kieckegereder et al. demonstrated that IDH mutation status is associated with a specific hypoxia/angiogenesis transcriptome signature predictable through perfusion MRI (83). Our results seem to confirm a role for perfusion-based analysis in discriminating IDH mutation, reflecting the known correlation with hypoxia inducible factor (HIF) and neoangiogenesis (84). Also, textural features achieved optimal results in the prediction of IDH mutation based on T1 images from NET (84.2%, ST classifier) and T2 images from CET (85.9%, AB classifier). The accumulation of D-2HG derived from IDH mutation induces epigenetic changes that lead to abnormal gene expression and impaired cellular differentiation, possibly contributing to intratumoral heterogeneity. Hsieh et al. demonstrated that textural features can differentiate IDH mutation with 85% accuracy in 39 patients with GBM. The Authors performed tailored biopsies demonstrating an agreement between prediction results and biopsy-proven pathology of 0.60 (85). Shape features of tumor necrosis demonstrated good accuracy for IDH mutation prediction in our model (**Figure S6D**). Such result may partly explain the relation between necrosis shape and survival as previously discussed (35, 77).

Ki-67 is a nuclear protein expressed by cells entering the mitotic cycle. In gliomas, the expression of Ki-67 is roughly proportional to the histologic grade, representing a proliferative index with prognostic correlation (86). Radiomic models predictive of Ki-67 expression have not been investigated before in the literature. In our analysis we achieved an accuracy of 86% for predicting Ki-67 expression through the AB. Intriguingly, best performing features were texture-based parameters extracted from the solid tumor (CET ROI) on ADC maps (**Figure S8**). These results perfectly agree with the role of Ki-67 as proliferative index in HGG, being ADC an MRI surrogate of cellularity (72, 73).

EGFR is a transmembrane tyrosine-kinase receptor for different growth factors, whose activation leads to DNA synthesis and cellular proliferation (87). Amplification of EGFR (especially EGFRvIII) is a common somatic mutation in HGG (4), with high relevance for the definition of GBM in the recent classification (6). Despite failure of initial attempts of targeting EGFR for therapy, the receptor remains of value for possible future treatments (87). In our results, EGFR showed best prediction performance with ST and AB classifiers. Particularly, rCBV features achieved a performance of 81% with AB classifier and T2 features achieved a performance of 77.8% with AB classifier on CET ROI. Highest scoring features were median intensity values for rCBV and textural features for T2 (**Figures S10A, B**). These results are supported by previous evidence. Hu et al. demonstrated a link between EGFR amplification and rCBV textural features, with correlation to microvessel volume and angiogenesis on tumor biopsies (88). Similarly, T2 textural features were shown to correlate to EGFR amplification (88).

Our study had some limitations. Firstly, even though ML studies in HGG often rely on limited populations (18, 19, 34, 36, 62, 77, 85, 88, 89), our sample size (156 patients) could be considered small. Nevertheless, our dataset includes clinical/genetic information (e.g., survival, MGMT, IDH, EGFR, and KI67), together with radiomic data from different MRI sequences (e.g., MPRAGE, FLAIR, ADC, rCBV, T1-wiethed, and T2-weighted), thus allowing us to combine information from different sources to better predict clinical and genetic variables. Due to the retrospective nature of the study, some sequences were not acquired for all the patients (**Table 1**). For this reason, prediction accuracy for each label was evaluated separately on each sequence, thus limiting performance bias. Moreover, some labels were not available for all the patients; consequently, the number of subjects split in train and test groups changed for each label-sequence combination. We tried to overcome this limitation by employing two well-known and effective techniques with the aim of balancing the asymmetric labels. Although undersampling of the majority class was considered a more effective approach in respect to an oversampling method (90), we decided to use SMOTE for unbalancing issues. As demonstrated in other SMOTE-based studies (24, 91), it could represent a suitable solution for our purposes. In order to overcome main SMOTE drawbacks (92, 93) we perform ML analysis with a significant number of cross-validations. Since we only split subjects into train and test groups, the lack of an additional validation cohort could represent a limitation of this study. To overcome this issue, we decided to report range of performance obtained applying four times stratified K-fold cross-validation. This approach provides a full accuracy range, which includes the results that an eventual validation test would produce.

## Conclusions

In the present study we were able to predict patient OS and highly relevant molecular features of HGG from preoperative MRI, comparing different ML classifiers. Ensemble classifiers (AB, ST, GB, and xGB) showed optimal performance in prediction tasks for all the studied variables. In particular, AB and xGB obtained maximum accuracy for survival, AB for IDH mutation, MGMT promotor methylation status and Ki-67 expression, and EGFR amplification. Ensemble learning outperformed classic ML algorithms in all tests, in agreement with previous literature. Best performing features from our analysis shed light on possible correlations between MRI and tumor histology, as well as molecular profiles and patient outcome in HGG. Our results may set a path for ML analysis standardization and clinical application. Future developments may include the evaluation of other genetic abnormalities, prediction of recurrence, and response to therapy.

## Data Availability Statement

The raw data supporting the conclusions of this article will be made available upon reasonable request to the authors.

## Ethics Statement

The studies involving human participants were reviewed and approved by Sant’Andrea Hospital, *via* Grottarossa 1035, 00189, Rome, Italy. Protocol Number: 19 SA_2020. The patients/participants provided their written informed consent to participate in this study.

## Author Contributions

LP and AN made substantial contributions to the conception and design of the work. LP, AN, ADN, FD, AV, VV, GR, and AS contributed to data acquisition and supervision. AN, ML, ET, and MR contributed to data analysis. LP, AN, ADN, MCR-E, AR, and AB contributed to data interpretation. LP, ML, and EM drafted the manuscript. All authors substantially revised the manuscript. All authors approved the submitted version.

## Funding

This study was supported by the grant “Progetti di Ateneo 2020” from La Sapienza University (Protocol ID: RP120172B9E252BD). Funding sources did not influence any phase of the present study.

## Conflict of Interest

The authors declare that the research was conducted in the absence of any commercial or financial relationships that could be construed as a potential conflict of interest.

The handling editor declared a shared affiliation with several of the authors, LP, FD, MCR-E, GR, AS, AR, ADN, AB, at time of review.

## Publisher’s Note

All claims expressed in this article are solely those of the authors and do not necessarily represent those of their affiliated organizations, or those of the publisher, the editors and the reviewers. Any product that may be evaluated in this article, or claim that may be made by its manufacturer, is not guaranteed or endorsed by the publisher.

## Supplementary Material

Supplementary Figure 1 Best results box-plots for Surv12 prediction among all sequences and ROI combination for all classifiers.

Supplementary Figure 2 First 15 significative features extracted from NET ROI on sequence ADC **(A)** and FLAIR **(B)** and from NEC ROI on sequence T2 **(C)** that provided best Surv12 predictions.

Supplementary Figure 3 Best results box-plots for MGMT prediction among all sequences and ROI combination for all classifiers.

Supplementary Figure 4 First 15 significative features extracted from CET ROI on sequence FLAIR that provided best MGMT predictions.

Supplementary Figure 5 Best results box-plots for IDH prediction among all sequences and ROI combination for all classifiers.

Supplementary Figure 6 First 15 significative features extracted from NET ROI on sequence rCBV **(A)** and T1 **(B)**, from CET ROI on sequence T2 **(C)**, and within NEC ROI on sequence T2 **(D)**, that provided best IDH predictions.

Supplementary Figure 7 Best results box-plots for KI-67 prediction among all sequences and ROI combination for all classifiers.

Supplementary Figure 8 First 15 significative features extracted from CET ROI on ADC sequence, that provided best KI67 predictions.

Supplementary Figure 9 Best results box-plots for EGFR prediction among all sequences and ROI combination for all classifiers.

Supplementary Figure 10 First 15 significative features extracted from CET ROI on rCBV **(A)** and T2 sequences, that provided best EGFR predictions.
