# Supplementary material for: AI and High-Grade Glioma for Diagnosis and Outcome Prediction: Do All Machine Learning Models Perform Equally Well?
Source: Front Oncol. 2021 Nov 23;11:601425. doi: 10.3389/fonc.2021.601425 (PMC8649764; doi:10.3389/fonc.2021.601425)
Supplement: Supplementary Figure 1 — Best results box-plots for Surv12 prediction among all sequences and ROI combination for all classifiers. [file DataSheet_1.zip › Supplementary_material/MRI_parameters.docx]

DETAILED ACQUISITION PARAMETERS

Patients acquired with the 1.5T scanner underwent the following protocol: axial T1-weighted spin echo (TR/TE, 600/12 ms; ST 4 mm; FA 150°; matrix 512 x 512), axial T2-weighted fast spin echo (TR/TE 2920/107; ST 4 mm; FA 180°; matrix 512 x 512), axial FLAIR (TR/TE 10000/126 ms; ST 4 mm, IT 2500 ms; FA 150°; matrix 512 x 512). DWI (TR/TE 3000/84 ms; ST 5 mm; FA 90°; matrix, 256 x 256) were acquired with three levels of diffusion sensitization (b-values 0, 500 and 1000). DSC images acquired during contrast injection (DOTAREM.; dose 0.1 mmol/kg, injection rate 4 ml/s) followed by a 20-ml saline flush, based on T2*- weighted gradient-echo echo-planar sequence (TR/TE 1490/40 ms; flip angle 90°; FOV 230 x 230 mm; matrix 128 x 128, 14 sections of 5 mm thickness, 50 volumes). MPRAGE (TR/TE 1840/4.4 ms; ST 1 mm; IT 1100 ms; FA 15°; matrix 256 x 256) after administration of contrast.

Patients acquired with the 3T scanner underwent the same protocol, with the following sequences: axial T1-weighted spin echo (TR/TE, 600/6.4 ms; ST 3 mm; FA 138°; matrix 512x512), axial T2-weighted fast spin echo (TR/TE 8694/148; ST 3 mm; FA 160°; matrix 512 x 512), axial FLAIR (TR/TE 7002/142 ms; ST 1 mm, IT 1892 ms; matrix 512 x 512). DWI (TR/TE 6500/88; ST 4 mm; FA 90°; matrix 256 x 256) were acquired with three levels of diffusion sensitization (b-values 0, 500 and 1000). DSC images acquired during contrast injection (DOTAREM.; dose 0.1 mmol/kg, injection rate 4 ml/s) followed by a 20-ml saline flush, based on T2*- weighted gradient-echo echo-planar sequence (TR/TE 2000/16 ms; flip angle 60°; FOV 230 x 230 mm; matrix 128 x 128, 20 sections of 4 mm thickness, 45 volumes). BRAVO (TR/TE 8.524/3.3 ms, ST 1 mm, IT 450 ms, FA 12°, matrix 256 x 256) after administration of contrast.
