# Supplementary material for: AI and High-Grade Glioma for Diagnosis and Outcome Prediction: Do All Machine Learning Models Perform Equally Well?
Source: Front Oncol. 2021 Nov 23;11:601425. doi: 10.3389/fonc.2021.601425 (PMC8649764; doi:10.3389/fonc.2021.601425)
Supplement: Supplementary Figure 1 — Best results box-plots for Surv12 prediction among all sequences and ROI combination for all classifiers. [file DataSheet_1.zip › Supplementary_material/Supplementary_tables.docx]

**Supplementary Table 1**. Surv12 Roc best results (reported as 95% confidence interval (CI))

| ROI | SEQ |  | xGB | GB | RF | LR | ST | KN | DT | AB | ST_ABC |
| --- | --- | --- | --- | --- | --- | --- | --- | --- | --- | --- | --- |
| NET | ADC | Roc % | (69,8-73,7) | (66,9-71,3) | (66,9-69,3) | (44-45,9) | (69,5-73) | (59-64) | (55,9-60,6) | (71,6-75,3) | (59,8-65) |
| NET | FLAIR | Roc % | (69,6-75) | (64,8-69,2) | (70,6-73,6) | (59,5-65) | (66,5-71,4) | (51-56,9) | (56,2-61,4) | (68-71) | (56,5-61,6) |
| NEC | T2 | Roc % | (71,9-76,3) | (62,7-67,2) | (63-69,8) | (55,9-61,7) | (71,3-75) | (49-55) | (57,5-61,8) | (70,5-74,4) | (53,4-59) |

**Supplementary Table 2**. MGMT Roc best results (reported as 95% CI)

| ROI | SEQ |  | xGB | GB | RF | LR | ST | KN | DT | AB | ST_ABC |
| --- | --- | --- | --- | --- | --- | --- | --- | --- | --- | --- | --- |
| CET | FLAIR | Roc % | (60,5-65) | (64-69,5) | (60,9-65,8) | (56,9-61,1) | (63,7-70,4) | (48,8-54,1) | (53-57,9) | (65,9-71,7) | (59-64,9) |

**Supplementary Table 3**. IDH Roc best results (reported as 95% CI)

| ROI | SEQ |  | xGB | GB | RF | LR | ST | KN | DT | AB | ST_ABC |
| --- | --- | --- | --- | --- | --- | --- | --- | --- | --- | --- | --- |
| NET | rCBV | Roc % | (80,6-85,7) | (79,3-84,7) | (75,2-81,4) | (75-80,9) | (83,4-88,3) | (65,3-72,6) | (75,3-81,3) | (84,3-89) | (79,5-84,5) |
| NET | T1 | Roc % | (76,4-82,4) | (77,8-83,7) | (75,8-80,7) | (65,6-70,1) | (80-85,9) | (62,5-71) | (73,4-79) | (82,8-88,7) | (77,3-82,6) |
| CET | T2 | Roc % | (78,6-83) | (78,7-83,8) | (78,9-83,8) | (57,2-62,6) | (79,6-83,9) | (68,6-74,6) | (74-79,6) | (80-84,6) | (79,8-84,2) |
| NEC | T2 | Roc % | (74,6-78,6) | (74,9-78,9) | (75,7-80,2) | (68,2-73,2) | (77-80,8) | (67-72,9) | (74,9-80) | (78,4-82,6) | (76,6-80,2) |

**Supplementary Table 4**. KI67 Roc best results (reported as 95% CI)

| ROI | SEQ |  | xGB | GB | RF | LR | ST | KN | DT | AB | ST_ABC |
| --- | --- | --- | --- | --- | --- | --- | --- | --- | --- | --- | --- |
| CET | ADC | Roc % | (61,7-67,6) | (61-67,9) | (63,7-71,3) | (47,3-54,3) | (59,7-66,8) | (56,7-62,9) | (56,3-63,8) | (65,3-72,9) | (61-67,8) |

**Supplementary Table 5**. EGFR Roc best results (reported as 95% CI)

| ROI | SEQ |  | xGB | GB | RF | LR | ST | KN | DT | AB | ST_ABC |
| --- | --- | --- | --- | --- | --- | --- | --- | --- | --- | --- | --- |
| CET | rCBV | Roc % | (60-67,8) | (60,9-68,3) | (60,7-68,6) | (57,8-66,5) | (61,9-69,4) | (58,8-68) | (54,7-64) | (70,8-77,8) | (58,6-66,7) |
| CET | T2 | Roc % | (65,9-75) | (65,7-73,6) | (72,9-79,7) | (62,2-68,5) | (65,3-74,4) | (56,3-64) | (51,6-59,8) | (70,6-77,6) | (61,5-69,7) |
