# Supplementary figures and images for: AI and High-Grade Glioma for Diagnosis and Outcome Prediction: Do All Machine Learning Models Perform Equally Well?
Source: Front Oncol. 2021 Nov 23;11:601425. doi: 10.3389/fonc.2021.601425 (PMC8649764; doi:10.3389/fonc.2021.601425)

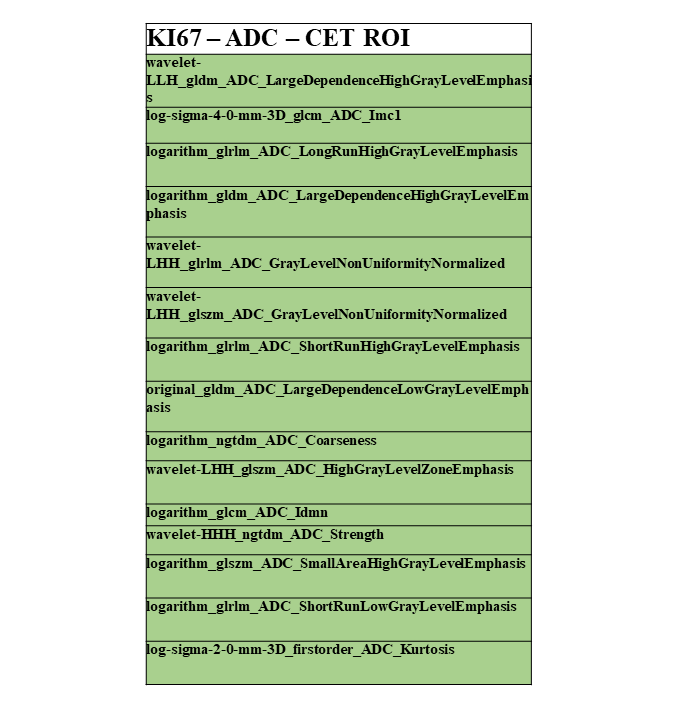

Supplement: Supplementary Figure 1 — Best results box-plots for Surv12 prediction among all sequences and ROI combination for all classifiers. [file DataSheet_1.zip › Supplementary_material/Figure S8.tif]

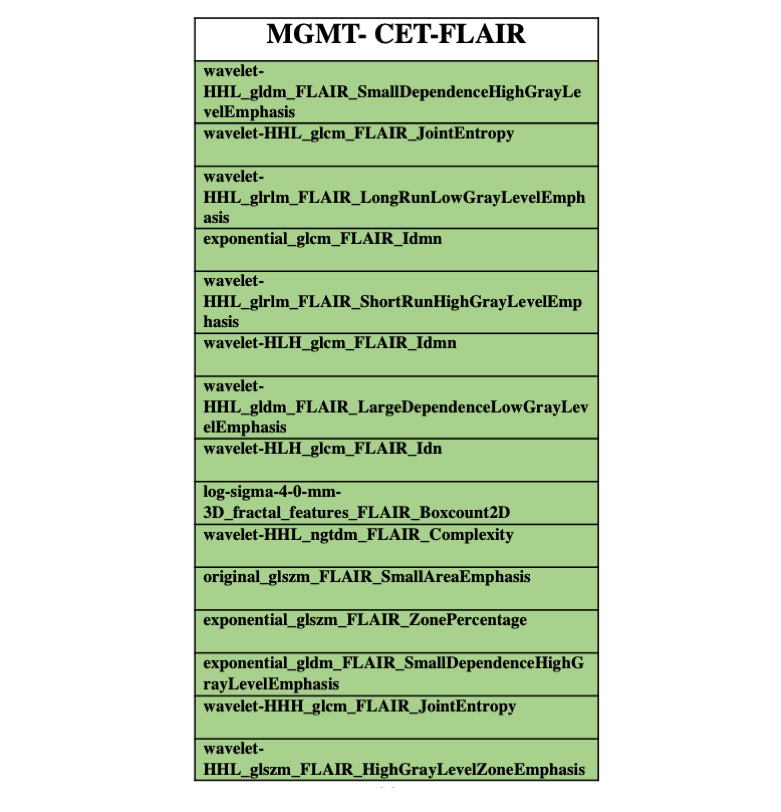

Supplement: Supplementary Figure 1 — Best results box-plots for Surv12 prediction among all sequences and ROI combination for all classifiers. [file DataSheet_1.zip › Supplementary_material/Figure S4.tiff]

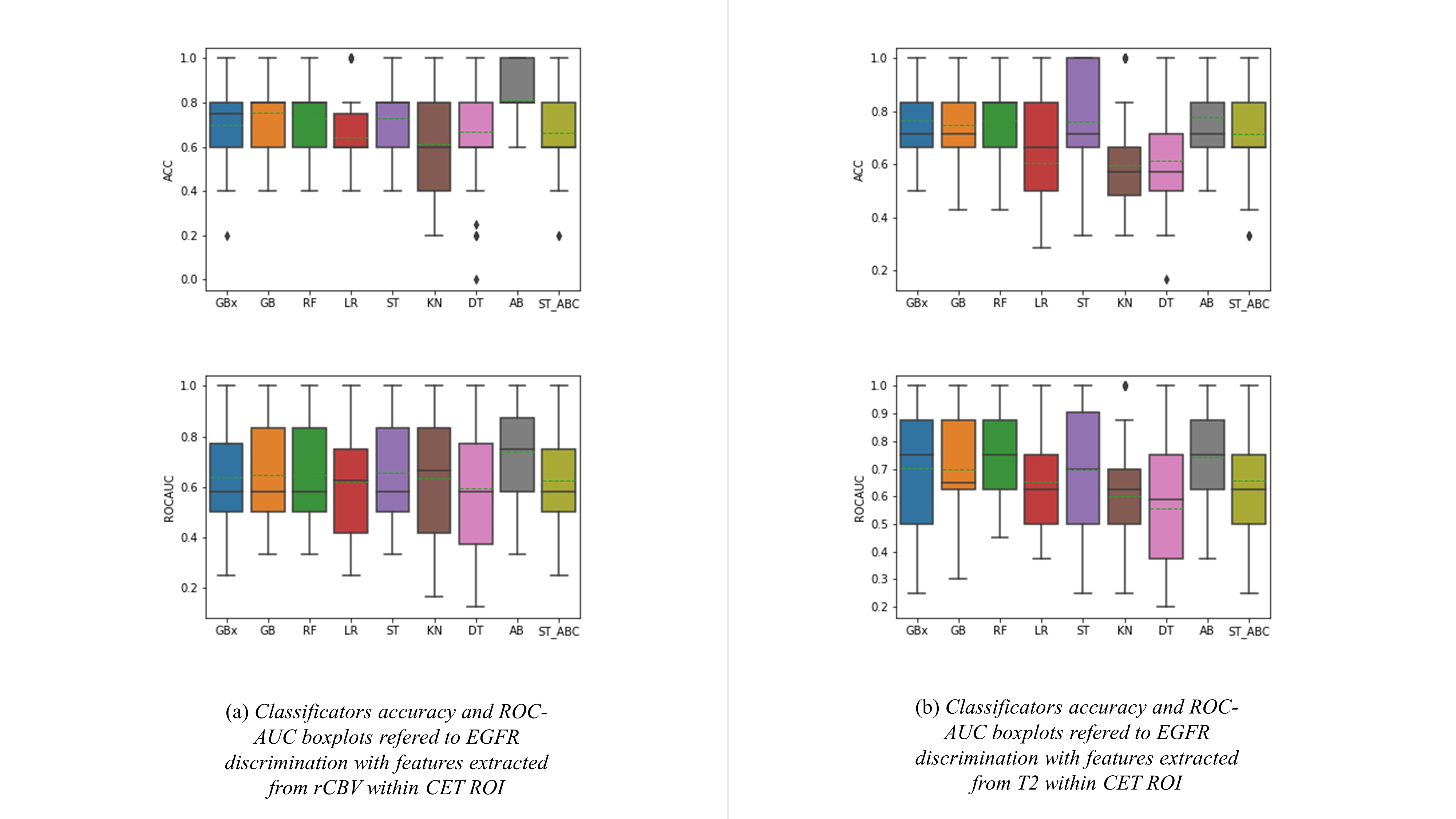

Supplement: Supplementary Figure 1 — Best results box-plots for Surv12 prediction among all sequences and ROI combination for all classifiers. [file DataSheet_1.zip › Supplementary_material/Figure S9.tif]

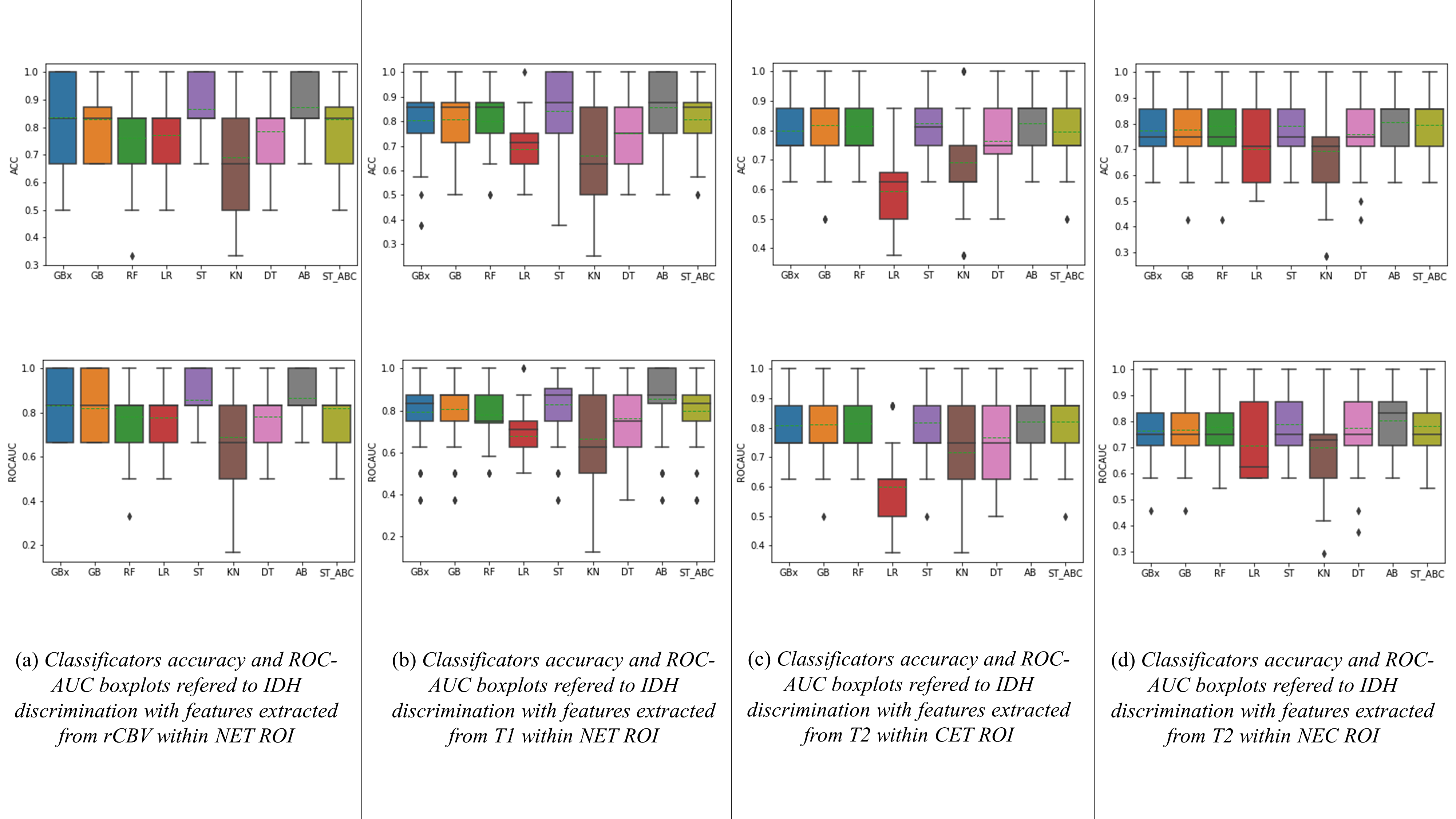

Supplement: Supplementary Figure 1 — Best results box-plots for Surv12 prediction among all sequences and ROI combination for all classifiers. [file DataSheet_1.zip › Supplementary_material/Figure S5.tif]

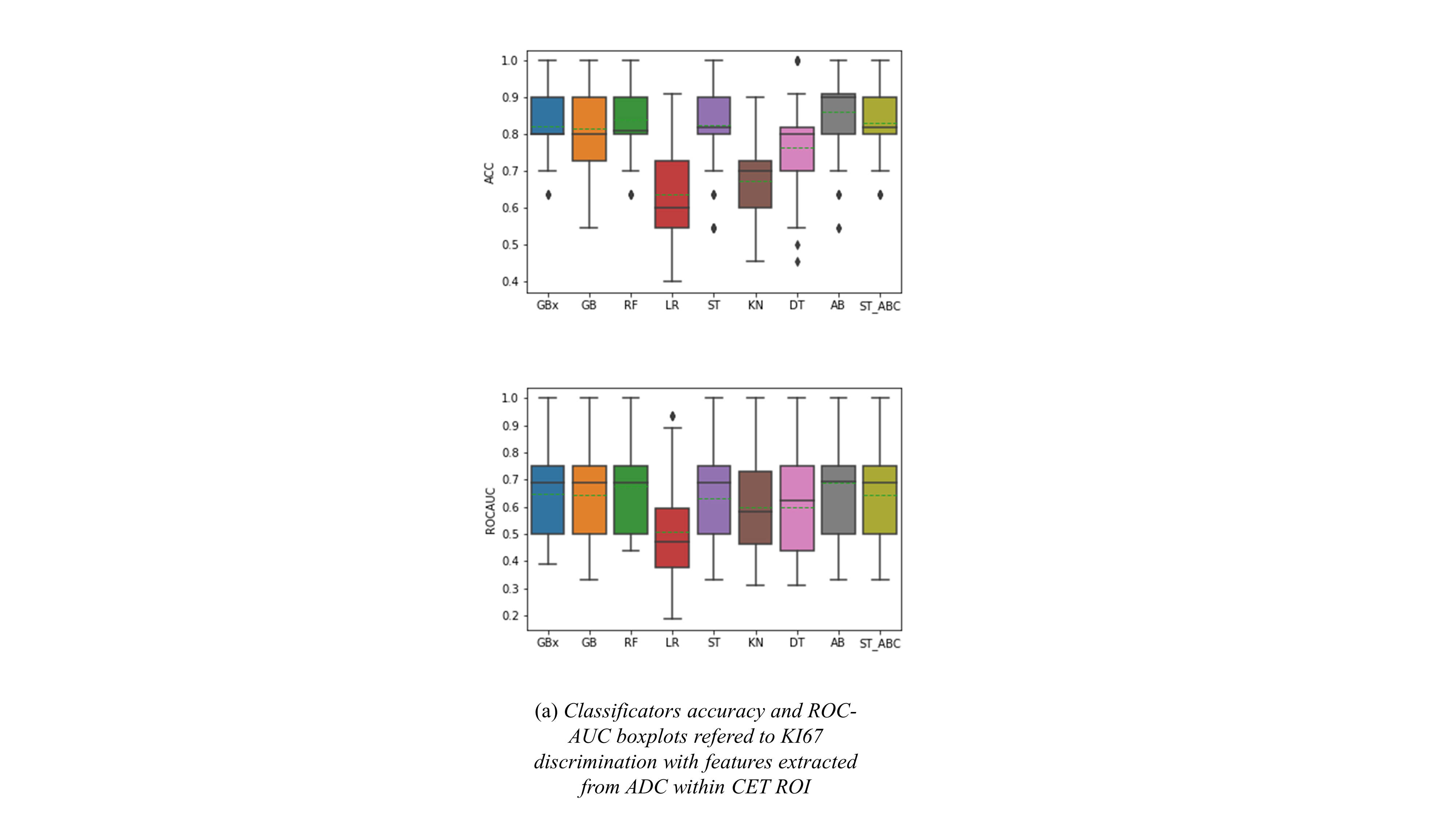

Supplement: Supplementary Figure 1 — Best results box-plots for Surv12 prediction among all sequences and ROI combination for all classifiers. [file DataSheet_1.zip › Supplementary_material/Figure S7.tif]

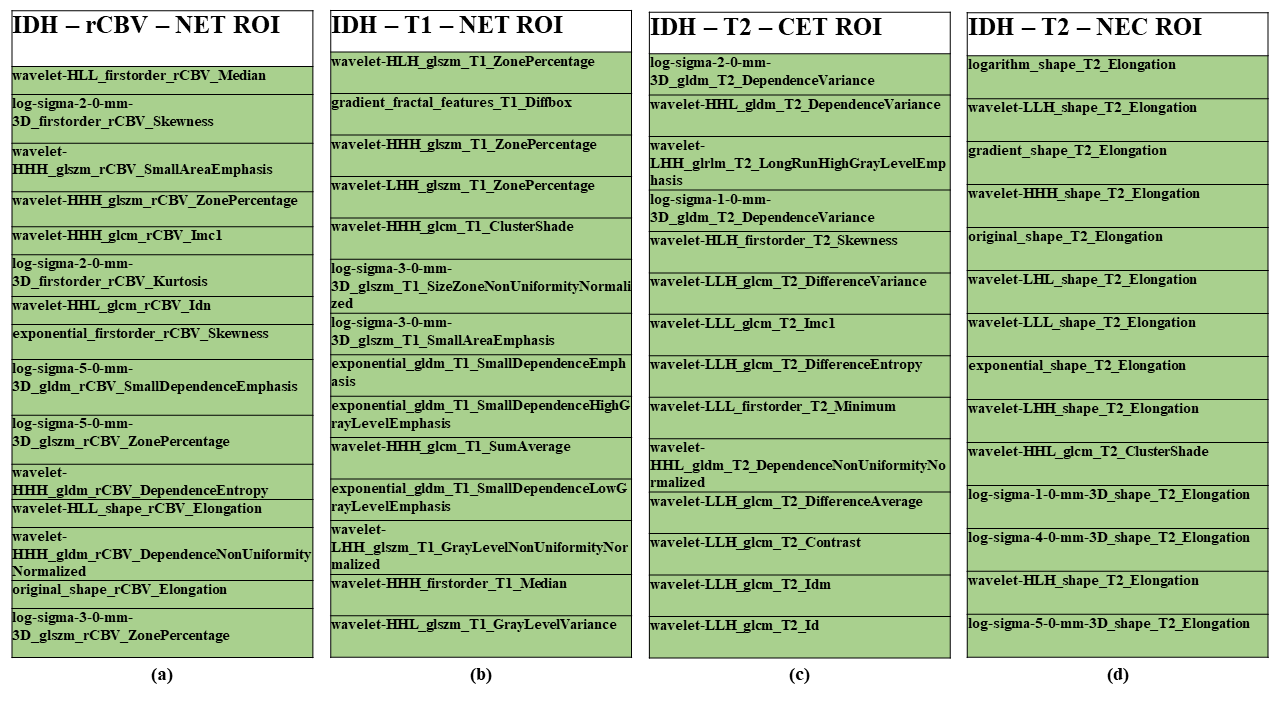

Supplement: Supplementary Figure 1 — Best results box-plots for Surv12 prediction among all sequences and ROI combination for all classifiers. [file DataSheet_1.zip › Supplementary_material/Figure S6.tif]

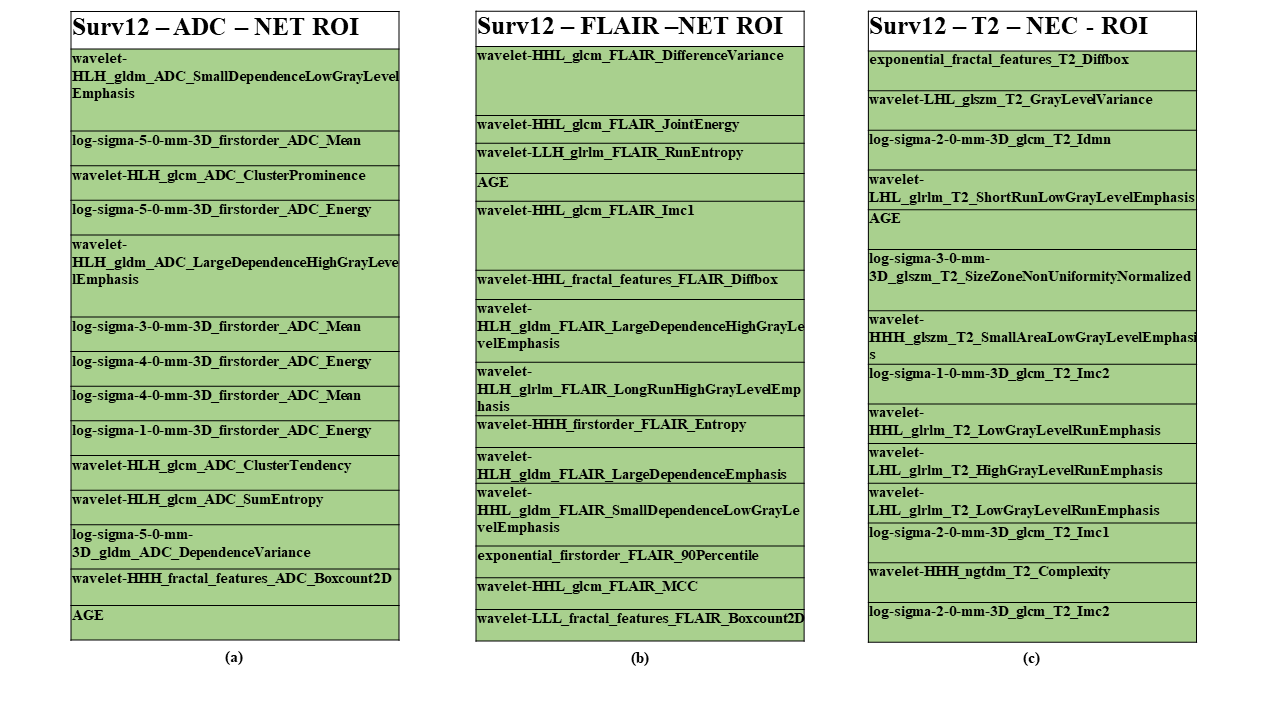

Supplement: Supplementary Figure 1 — Best results box-plots for Surv12 prediction among all sequences and ROI combination for all classifiers. [file DataSheet_1.zip › Supplementary_material/Figure S2.tif]

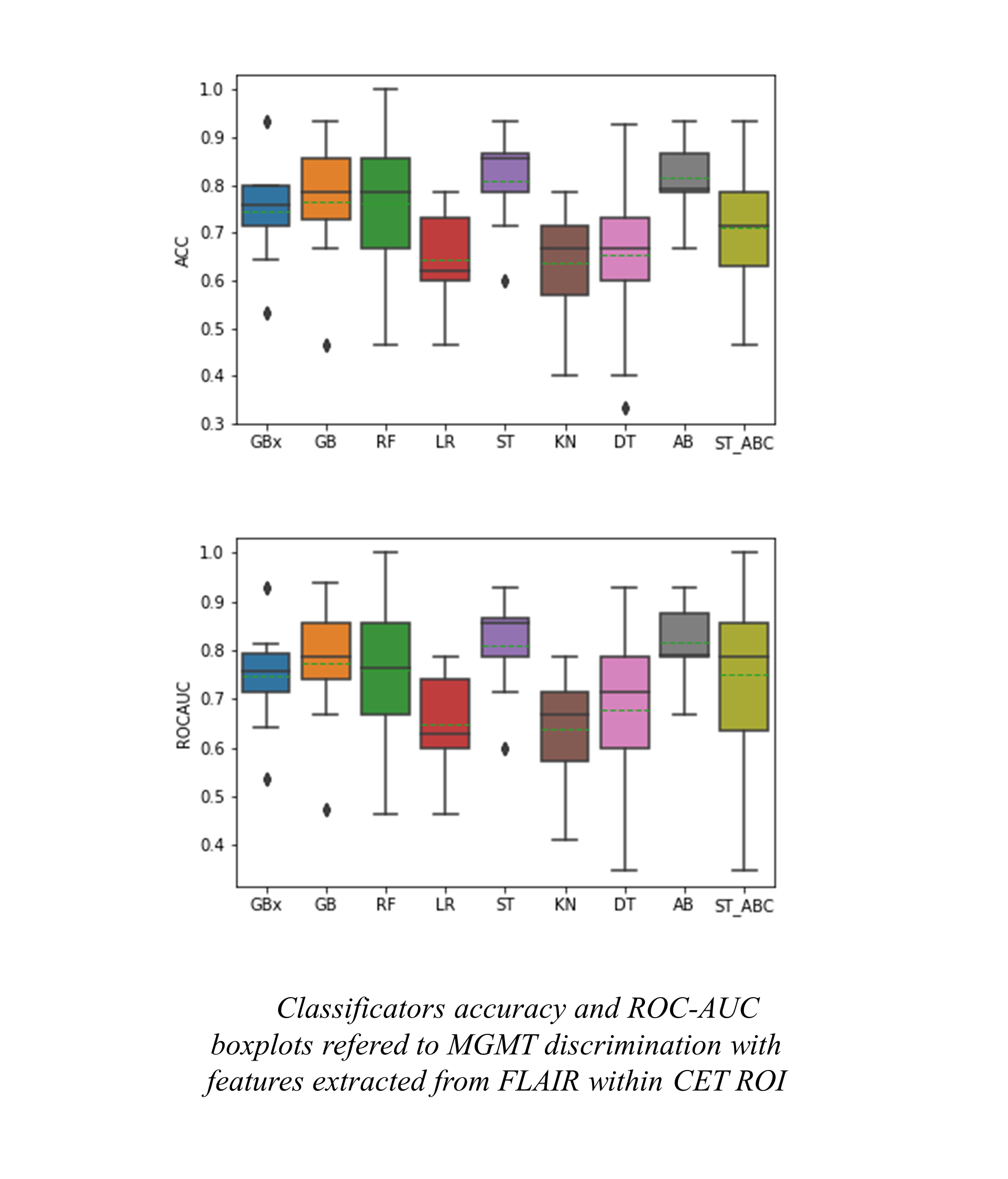

Supplement: Supplementary Figure 1 — Best results box-plots for Surv12 prediction among all sequences and ROI combination for all classifiers. [file DataSheet_1.zip › Supplementary_material/Figure S3.tif]

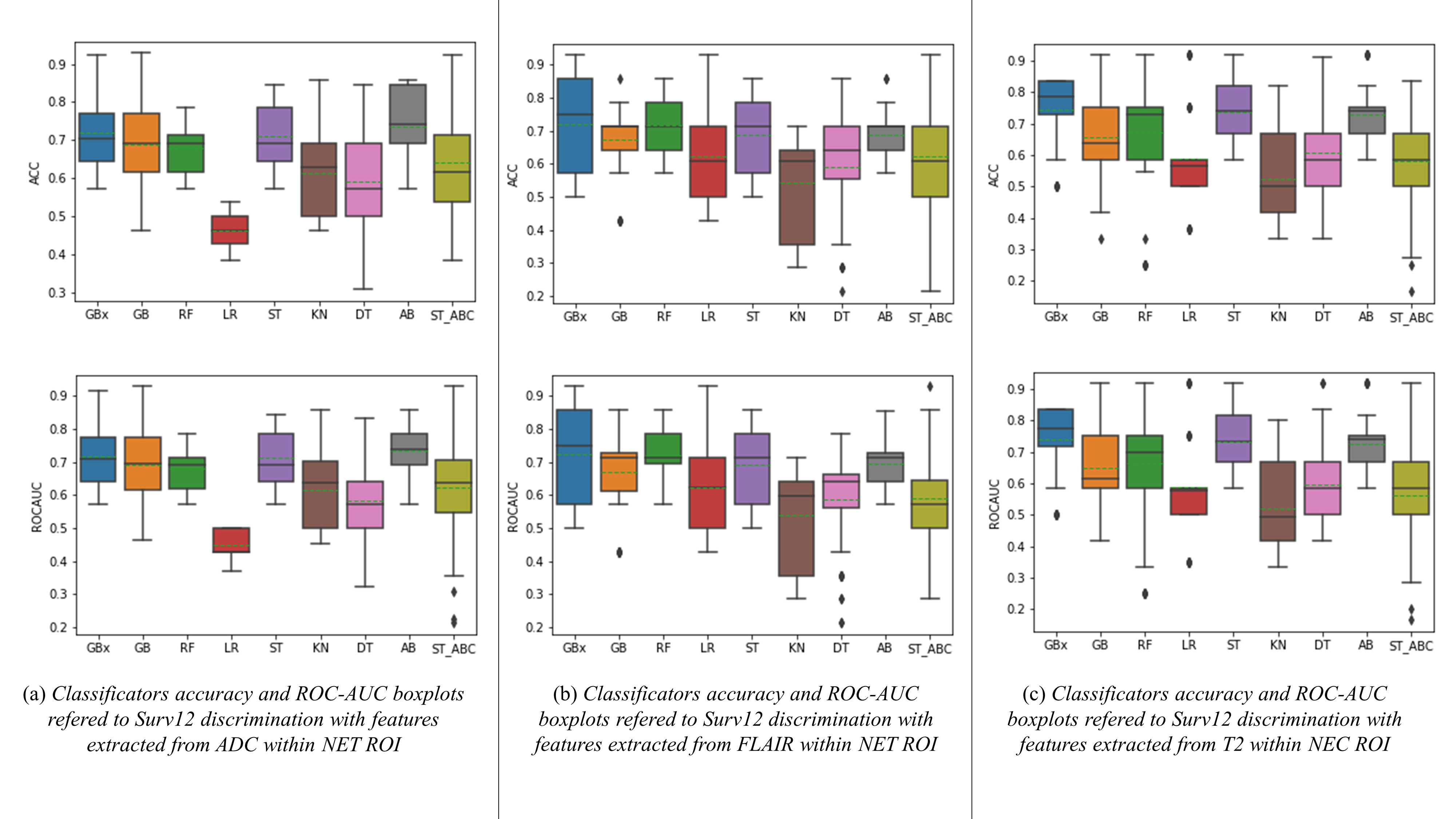

Supplement: Supplementary Figure 1 — Best results box-plots for Surv12 prediction among all sequences and ROI combination for all classifiers. [file DataSheet_1.zip › Supplementary_material/Figure S1.tif]

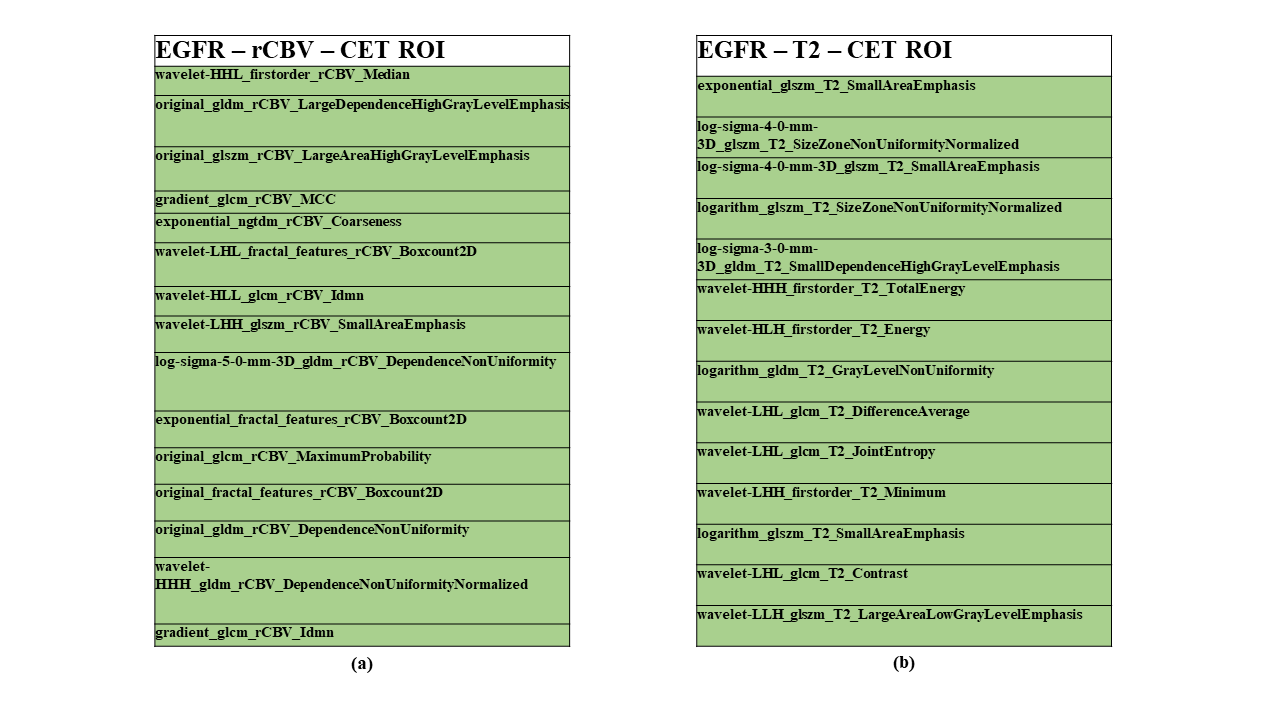

Supplement: Supplementary Figure 1 — Best results box-plots for Surv12 prediction among all sequences and ROI combination for all classifiers. [file DataSheet_1.zip › Supplementary_material/Figure S10.tif]
